# Survey of e-learning implementation and faculty support strategies in a cluster of mid-European medical schools

**DOI:** 10.1186/s12909-015-0420-4

**Published:** 2015-09-03

**Authors:** David Alexander Back, Florian Behringer, Tina Harms, Joachim Plener, Kai Sostmann, Harm Peters

**Affiliations:** 1 Department of Traumatology and Orthopedics, Bundeswehr Hospital, Scharnhorststrasse 13, 10115 Berlin, Germany; 2 Dieter Scheffner Center for Medical Education and Research, Charité – Universitätsmedizin Berlin, Charitéplatz 1, 10117 Berlin, Germany

## Abstract

**Background:**

The use of electronic learning formats (e-learning) in medical education is reported mainly from individual specialty perspectives. In this study, we analyzed the implementation level of e-learning formats and the institutional support structures and strategies at an institutional level in a cluster of mid-European medical schools.

**Methods:**

A 49-item online questionnaire was send to 48 medical schools in Austria, Germany and Switzerland using SurveyMonkey®. Data were collected between February and September of 2013 and analyzed using quantities, statistical and qualitative means.

**Results:**

The response rate was 71 %. All schools had implemented e-learning, but mainly as an optional supplement to the curriculum. E-learning involved a wide range of formats across all disciplines. Online learning platforms were used by 97 % of the schools. Full-time e-learning staff was employed by 50 %, and these had a positive and significant effect on the presence of e-learning in the corresponding medical schools. In addition, 81 % offered training programs and qualifications for their teachers and 76 % awarded performance-oriented benefits, with 17 % giving these for e-learning tasks. Realization of e-learning offers was rewarded by 33 %, with 27 % recognizing this as part of the teaching load. 97 % would use curriculum-compatible e-learning tools produced by other faculties.

**Conclusions:**

While all participating medical schools used e-learning concepts, this survey revealed also a reasonable support by institutional infrastructure and the importance of staff for the implementation level of e-learning offerings. However, data showed some potential for increasing tangible incentives to motivate teachers to engage in further use of e-learning. Furthermore, the use of individual tools and the distribution of e-learning presentations in various disciplines were quite inhomogeneous. The willingness of the medical schools to cooperate should be capitalized for the future, especially concerning the provision of e-learning tools and concepts.

**Electronic supplementary material:**

The online version of this article (doi:10.1186/s12909-015-0420-4) contains supplementary material, which is available to authorized users.

## Background

In parallel with the technological revolution of the past decades, digitally supported learning tools and formats (e-learning) have become an important component in many medical curricula [1]. An increasing number of e-learning tools has been developed and is now employed in various settings according to the subject and intention of the educational endeavor [2–5]. Today´s medical students have grown up mostly in an already technologically supported learning environment. In general, they positively rate the integration of e-learning [6–9] and e-learning is frequently integrated with face-to-face teaching as blended learning format [10]. The production of e-learning materials is often time-consuming and competes with the more and more compressed work-schedules of medical doctors and their limited time resources [11]. In addition, most teachers need technical and expert support when it comes to the production and implementation of e-learning [12].

International recommendations state that a faculty-wide use of electronic learning scenarios should be a central part in the strategic development of medical programs [13]. In the literature, there are a number of reports on e-learning in medical education, but mainly from discipline-specific perspectives [2, 4, 9]. Analyses and research on the faculty’s application of e-learning formats and supporting infrastructure in medical schools can only rarely be found [14]. Medical faculties can take hold of a wide range of potential structures and strategies to facilitate and foster the use of e-learning in their medical curricula. These range from the provision of *Learning Management Systems* (LMS) as internet-based software programs for the delivery and tracking of e-learning across a medical school [1] authoring tools, up to permanent e-learning staff, qualification programs and training courses for the implementation of e-learning [15], quality assurance programs for the production and implementation of e-learning [16, 17], funding of e-learning projects and performance-orientated payments or awards [18, 19].

The aim of this study was to systematically analyze the implementation level for e-learning in medical education from a medical school institutional perspective. We took advantage of an existing working group “*New Media*” within the *Gesellschaft für Medizinische Ausbildung* (*Society for Medical Education*) that brings together representatives from medical schools of the three mid-European countries Austria, Germany and Switzerland [20]. In addition to the level of implementation, we analyzed the currently operating institutional support structures and strategies for the provision of e-learning and asked for e-learning issues that should be approached in the near future.

## Methods

The target group of the survey consisted of all existing 48 medical schools in Austria (*n* = 4), Germany (*n* = 36) and Switzerland (*n* = 8). Contact persons of the survey were members of the working group *New Media* of the *Society for Medical Education* and faculty members at the deaneries of student affairs responsible for e-learning. An item-based questionnaire was constructed on the bases of literature search and according to methods of empirical social research [21]. The items were grouped into the following three main areas:

- General information about the medical school and provision of e-learning
- Information about the infrastructural conditions of e-learning supporting measures
- Information about requirements of and incentives for staff in the field of e-learning

The initial questionnaire was validated through a peer review approach online according the pre-test method [22] during the winter semester 2012/13. Peer reviewers (*n* = 4) were both faculty staff members responsible for e-learning in their medical school, and members of the *Society for Medical Education* [20]. The returned comments were collected and the questionnaire adapted accordingly. The local responsible ethical committee of the university was informed about this survey and gave its written consent (Ethikkommission Charité - Universitätsmedizin Berlin, Ethikausschuss 4, Campus Benjamin Franklin, Berlin, Germany). The survey followed the directives of the Helsinki declaration.

The final questionnaire for this study contained a total of 49 items, with 27 closed questions and 22 questions for free text answers (Additional file 1). The questionnaire was delivered to the 48 medical schools in Austria, Switzerland and Germany using the online program SurveyMonkey® (SurveyMonkey, Oregon, USA). Access to the questionnaire was provided by an email link. Through electronic pre-determination of the response options supplied by SurveyMonkey®, users were offered the option of skipping or omitting certain questions. The survey started in February 2013 and after 4 reminders, data collection ended in September 2013. Reminders consisted of reminder emails to the correspondent involved. In cases where an answer was still missing, we tried to contact assistants of the deaneries of student affairs by email. In a last attempt the deanery was called by phone.

Quantitative analysis was undertaken where results of the closed questions (mainly yes/ no) were expressed as percentage of their relative occurrence per item and were calculated by the SurveyMonkey® program. Spearman´s rank-order correlations were applied by using the SPSS® 17.0 statistics software (SPSS Inc., Chicago, USA) in order to analyze potential coherences between the indicated size of a medical school and its financial means or the number of employees for an e-learning department. Additionally, the chi-square test was used to analyze if the presence of e-learning staff predicted the use of any modalities, indicated by the medical schools in the closed questions. Alternatively, the Mann–Whitney *U* test was performed to analyse the implementation level of e-learning in the existing disciplines at the medical schools, and that of e-learning tools both in general and in dependence of the presence of e-learning staff.

The individual responses to the free text were quantitatively and independently analyzed by two of the authors for repetitive themes and then summarized.

## Results

A total of 34 out of the 48 online questionnaires was returned (71 %). After analysis of all data, seven questions for free text answers were excluded due to a low return rate (n ≤ 5) or due to ambiguous responses (Additional file 1). The responses (16 entries) came from e-learning staff (*n* = 8), dean’s office (*n* = 4) and quality management, exam coordination, IT and didactics (*n* = 1 each). The sizes of the various institutions varied from 100–500 students (*n* = 1), 500–1500 (*n* = 6), 1500–3000 (*n* = 19), 3000–6000 (*n* = 4), up to 6000–9000 students (*n* = 2). Nine universities voluntarily provided their names (seven German, one Austrian and one Swiss).

**Fig. 1:**
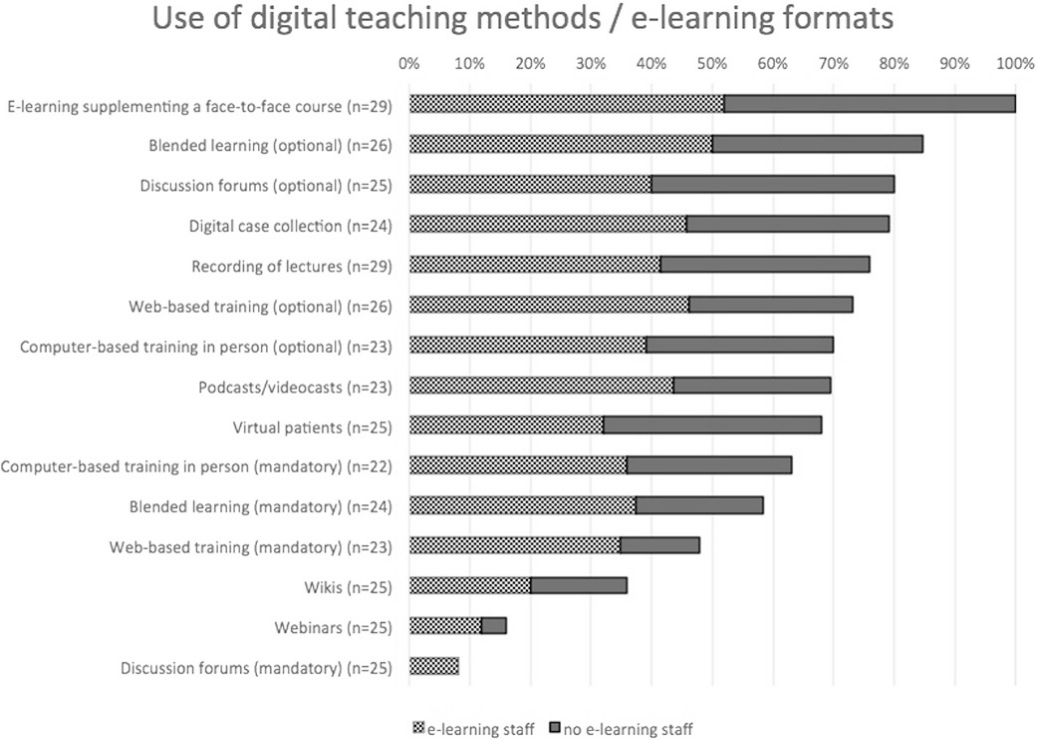
Relative use of various e-learning formats at medical schools. The numbers of medical schools answering these items are shown in brackets. The graph shows the relative implementation level of each item in relation to where e-learning staff was present or not. There was a statistically significant higher implementation level of the provided total amount of e-learning items when e-learning staff was present (35.9 +/−13.1 %) versus not present (26.1 +/−13.3 %) in the medical school (*p* < 0.05, Mann–Whitney *U* test)

**Fig. 2:**
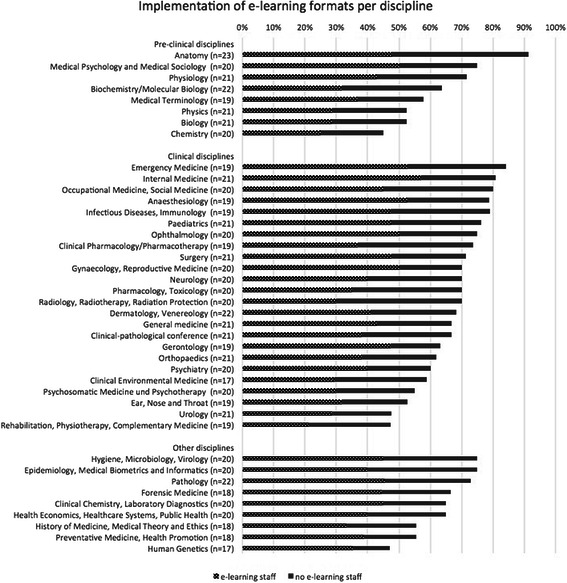
Relative distribution of e-learning offerings in various disciplines at medical schools. The numbers of the medical schools answering these items are shown in brackets. The main question asked was: “Which of the following disciplines at your medical school offer e-learning?” (Individual disciplines and their grouping into preclinical/clinical/ other disciplines were given). The graph shows also the relative implementation level of all disciplines in relation to where e-learning staff was present or not. There was a statistically significant higher implementation level of the general existence of e-learning offers in the individual disciplines when e-learning staff was present (40.0 +/−8.5 %) or not (26.2 +/−6.6 %) in the medical schools (*p* < 0.001, Mann–Whitney *U* test)

General information about the provision of e-learning at the faculty level is given in Table 1. At all medical schools, e-learning was an integrated element in their undergraduate medical education curricula. The majority of e-learning tools are used as optional tools for students’ education. Between 60 and 70 % of the medical schools answering used blended learning formats or computer-based trainings as a mandatory part of their curriculum. While an optional use of discussion forums was indicated by more than 75 %, mandatory integration of this tool was noted by less than 10 %. Figure 1 shows the relative distribution of various e-learning formats with high levels for e-learning as an addition to face-to-face teaching, and low levels for the use of Wikis and Webinars. Figure 2 documents the percentage of e-learning offerings in various disciplines. There were no 100 % or 0 % scores. High scores were found for Anatomy, Internal Medicine and Emergency Medicine, while Chemistry, Human Genetics and Gerontology had relatively low scores.

**Table 1:** General information about e-learning provision at medical schools (*n* = 34; results are expressed as percentage of total answers per item). The symbol * indicates a significant difference between medical schools with (34.4 %) and without e-learning staff (9.4 %) with a *p* < 0.01 (chi-square test of independence)

Question	Yes (%)
General information about e-learning at the medical schools	
Do e-learning tools for the education of students exist at your medical school?	100 %
- predominantly mandatory	9.4 %
- predominantly optional	90.6 %
Is there a specific set of recommendations for the application of e-learning by your medical school from the academic board or deanery?	43.8 % *
Do they stipulate the use of a quality assurance code for your e-learning activities?	
- If so, are these quality criteria designed to comply with the standards stipulated by the German Medical Association or similar (please see the PDF file attached for details)	48.4 %
	20.0 %
Is there a faculty-wide strategy for increasing the scope and quality of e-learning tools over the coming years in your medical school?	58.1 %
Do you take gender criteria into account when developing e-learning activities?	39.3 %
Do you wish more support from relevant discipline societies (GMA/GMDS etc.) and academic institutions or public agencies (DFG/BMBF/State Ministries) for the development of e-learning activities?	75.9 %

On the average, medical schools had two permanent staff members working on e-learning (median 1, range 1–5), as well as three assisting students (varying from 0–4 at most places, whereas one university reported eight, and another one 23). Fifty percent employed permanent staff almost exclusively in charge for e-learning (Table 2). The budget for the implementation of e-learning each year varied from “no budget” (*n* = 5) through €10,000–50,000 (*n* = 4) and €50,000–100,000 (*n* = 2) up to €100,000– 250,000 (*n* = 2). There was a weak negative correlation trend that was not statistically significant for the size of the medical school and their financial means (r(10) = - 0.39, *p* = 0.20). A weak positive correlation trend which was not statistically significant could be observed for the size of medical school and the number of employees within an e-learning department (r(14) = 0.17, *p* = 0.53).

**Table 2:** Infrastructural information about e-learning provisions at medical schools (*n* = 34; results are expressed as percentage of total answers per item). The symbol * indicates a significant difference between medical schools with (44.8 %) and without e-learning staff (17.2 %) with a *p* < 0.01 (chi-square test of independence)

Questions	Yes (%)
Infrastructural information about the provision of e-learning	
Does your medical school offer…	
a) … Performance-orientated financial rewards (LOM)?	75.9 %
b) … Specific LOM for teaching?	66.7 %
c) … Is e-learning associated with the award of LOM?	16.7 %
Do you have permanent staff in your medical school who is employed to deal with e-learning (an e-learning team/department)?	50.0 %
Do you offer training or qualification programs for teachers…	
a) … that deals directly with the authoring systems of programs in use at your medical school?	80.7 %
b) … on the topic of e-learning (general information)?	70.0 %
c) Would you make use of training programs that have been developed at other universities for the training of your teachers?	84.6 %
Does your medical school use electronic means to carry out summative (mandatory) exams?	53.3 %
Does your medical school also offer e-learning formative exams to students?	58.6 %
Do you reward your teachers in some form for…	
a) … the development of e-learning tools/courses?	32.1 %
b) … the implementation of e-learning tools/courses?	33.3 %
Do development and implementation of e-learning tools/courses count towards teaching activities or load?	24.1 %
- If not, is this planned at your medical school for the future?	39.1 %
Would you consider offering e-learning opportunities developed by other medical schools for inclusion in teaching by your medical school, if they fitted into the curriculum?	96.6 %
Are teachers at your medical school encouraged (through instructions, study regulations, etc.) to prepare e-learning tools?	56.7 %
Do you regularly evaluate the opinions, attitudes and experiences of your teachers on the subject of e-learning in your medical school?	31.0 %
Do you regularly evaluate the opinions, attitudes and experiences of your students on the subject of e-learning in your medical school?	62.1 % *
Does your medical school or do your students recognize outstanding e-learning opportunities with awards?	13.3 %

Concerning the impact of the presence of e-learning staff, a significant correlation could be shown for the existence of recommendations for the application of e-learning by a medical school (Table 1). Also, the evaluation of students on the subject of e-learning was performed significantly more often when e-learning staff was present (Table 2). There were also significantly higher implementation levels of the provided total amount of e-learning items (Fig. 1), as well as of the general existence of e-learning offers in the disciplines of medical schools (Fig. 2).

Information about the infrastructural support for e-learning provision by the medical schools is given in Table 2. Out of the responding medical schools, 81 % (*n* = 26) used a single LMS, 16 % (*n* = 5) used more than one and only 3 % (*n* = 1) did not use any at all. Twenty-five medical schools provided details of the programs they used for the creation of e-learning contents and 20 listed programs that they used for the recording of lectures (see Table 3). Special rewards associated with the commitment of teachers to e-learning provision differed in between the medical schools (11 responses). The most common rewards were performance-based grants (*n* = 6) and the awarding of prizes (*n* = 5) such as “Teacher of the Year”. Furthermore, support was provided with student assistants or specific teaching projects for e-learning. Two medical schools included requirements for the development of e-learning resources as part of their tenure scheme. Eight medical schools reported about certification schemes associated with the preparation and implementation of e-learning tools. The following solutions were put forward: summary on the basis of semester hours as part of the teaching responsibility (*n* = 3), regular implementation as a learning format in the course regulations, tenure scheme regulations or federal state teaching regulations (*n* = 1 for each). Two schools did not name any particular arrangement.

**Table 3:** Software programs used for the creation of e-learning contents recording of lectures. The questions were: “What is/are the name/s of the LMS you are using at your medical school?”, “Which programs do you offer your teachers for the development and creation of e-learning contents?”, “Which programs are used alongside those you offer by your teachers on their own initiative (as far as you know of?)”, “If you record lectures—which tools do you use?”

Program type	Product examples
Authoring systems	Docendo, Mediabird, Mediasite, Articulate, Lectora
	Wondershare (4x), Mediator Authoring Software
Case-based learning systems	CAMPUS (4x), Casus (3x), Inmedea Simulator, Casetrain
Learning management systems	Moodle (14x), Ilias (12x), OLAT (3x), Stud.IP (3x), Blackboard (2x), ALMA WEB (1x), Metacoon (1x), MedPol (1x), ILKUM (1x)
Microsoft Office Products	Word, Excel, PowerPoint, Access
Apple Products	iTunes U, Onyx Mac
Other products	Adobe Collection (Acrobat, Photoshop, Premiere, E-Learning Suite, CreativeSuite) (2x), Raptivity, Zoomify Image Viewer, Primal Pictures, Mediscript Online, Camtasia Studio (11x), WebKit
Programs for the recording of lectures	Camtasia Studio (10x), Lecturnity (3x), Adobe (Adobe Connect, Adobe Premiere) (3x), WOWZA Streaming Server, RUBcast, Vilea, LifeSize

With regards to a question about challenges for the future in the field of e-learning, the 21 responses (Additional file 2) could be crystallized into the following themes:

1. Improvement of the infrastructure and implementation in the curriculum (especially blended learning; reinforced mandatory, less optional use)
2. Creation of a faculty-wide strategy on e-learning
3. Improvement of personal resources and general financing
4. Construction of a system for teachers to encourage the creation of e-learning opportunities, as well as improved integration of teachers into e-learning concepts
5. Intensification of training measures for teachers in e-learning
6. Increased incorporation of smartphones, tablets and social media
7. Improvement of e-learning opportunities (portfolio extension, more interactivity and realism, research on benefits of e-learning and new potentials)
8. Clarification of copyright issues

## Discussion

The survey presented here showed that e-learning has reached a high implantation level in the cluster of analyzed mid-European medical schools. However, the use of single tools and the distribution of e-learning offerings in individual disciplines were seen to be inhomogeneous among the institutions. The results also highlight that there is a potential for improvements regarding motivational incentives for the teaching staff which is dealing with the development and implementation of e-learning scenarios.

Concerning the level of e-learning implementation, it can be stated that all of the addressed schools did use e-learning proposals to some extent. E-learning is also established in its basic use as a means to offer support to classic teaching of face-to-face courses. Among these offers, non-collaborative formats (such as podcasts or lecture recordings) are predominantly used as mandatory supplements. Future developments should consist of an integration of mandatory collaborative e-learning tools as a holistic approach that includes digitally driven assessment-formats as well [23]. In this study, gender issues were taken into account for developing e-learning activities in general by less than half of the participating medical schools. The survey did not go into any details, for instance into the use of gender-neutral language, avoiding gender stereotypes or gender medicine as a topic itself.

Among such methods of teaching, many formats seem to be well-known and widely implemented, while tools that focus on online collaboration are less presented especially as mandatory didactic elements. As there can be found successful usage of these—like that of wiki—in the literature [24], it can be assumed that their implementation depends both on the familiarity of teachers with the tools as curricular implementation and instructional design of the course [25].

There was an inhomogeneous representation of e-learning offers for the single medical disciplines in this survey. However, the individual number of medical schools which provided offers was also inhomogeneous for every particular discipline, an observation which has already been reported e.g. for radiology [26]. The presented results suggest that there is a lower level of implementation in smaller disciplines, such as otorhinolaryngology or human genetics. Here, implemented examples of successful projects in small disciplines [27] can be seen as great potential for sharing successful formats between medical schools in the future.

Regarding infrastructure and staff, all participating medical schools had faculty members as responsible contact persons for e-learning matters. For the technological infrastructure it can be stated that LMS were firmly established, with open source platforms predominantly used by the majority of the participants [1]. It is remarkable that the majority of the medical schools observed here preferred an in-house open source model to outsourcing their LMS. Although the use of open source systems is related to altered in-house costs, this might be acceptable to realize a higher flexibility in customizing the systems to the medical school’s needs [28]. Consequently, it can be assumed that a high proportion of the named e-learning staff capacities are allocated to host the LMS. However, it could also be shown that the existence of e-learning staff is positively correlated with the presence of e-learning offers at a medical school. This stresses the use of employing personnel merely dedicated to managing a medical schools’ e-learning portfolio.

Teachers could make use of different programs for the development of contents in the context of their didactic teaching scenarios, depending on the medical school. The vast majority offer trainings or qualification programs for teachers. That this is important was also postulated by Cook and Triola as well as by Kowalczyk and Copley, who stressed the need for faculty development in the use of current tools and ongoing training in emerging technologies [29, 26].

Alongside existing infrastructural services, motivational incentives will be important to encourage especially clinical teachers to deal with the provision of e-learning offers, as this is often time and resource-intensive [30]. In the study presented here, less than one-third of the participating medical schools rewarded the creation or implementation of e-learning tools/courses, and less than one fifth awarded funds or prizes for tasks undertaken in the field of e-learning. A further problematic aspect was that an evaluation of opinions, attitudes and experiences of teachers who are involved in the development of e-learning seems rarely to be taken into account [6]. Although a small proportion of participating centers recognized the implementation of e-learning activities as an additional achievement when looking at admission to a tenure scheme, a larger number planned to adopt their practices in this field—a promising approach, as already reported in the literature [31].

Quality assurance protocols for e-learning scenarios [16] were in place at only a handful of medical schools. As such a standardization is intended to ensure the maintenance of medical standards and the attainment of curricular learning goals [17], addressing this backlog would help to create certified e-learning tools which could be of use for a larger number of medical schools [27].

Altogether, the findings of this study can be seen as part of a detailed strategy of most of the medical schools to increase the scope and quality of their e-learning programs in the next years. Future challenges for a wide use and cooperation in e-learning aspects among medical schools can in fact build on the high willingness of the medical schools to use and exchange e-learning offerings of other universities when it comes to the education of students and also the training of teachers themselves. An interesting approach for the future might here be the development of a central—maybe even international—database of e-learning contents for medical schools, where an exchange or common use of data could take place [17, 27]. The majority of the faculties stated that professional societies, academic institutions or ministries should support the development of e-learning activities. Here, an inter-faculty discussion platform supported by these institutions could be helpful to answer many of the questions that the individual medical schools have in the field of e-learning—e.g. copyright or financial issues or the establishment of blended learning concepts.

Limitations of the validity of the present documentation which must be highlighted include the finding that responses were not collected from all of the medical schools addressed. Therefore, the results shown here provide insight into the current status of the field of e-learning but cannot be used for definitive statements. This could also contribute to a bias that potential regulatory differences in curricular uses of e-learning between individual countries and federal states were not captured by this survey. In addition, the option of leaving certain questions blank meant that there was variation in the numbers of answers collected. Also, the faculty positions of the addressees were seen to be inhomogeneous, and this may have influenced their understanding and perceptions as a confounding variable in answering the questions. Finally, the questions used can only provide a brief representation of the different situations in individual disciplines at the various universities.

As this evaluation focused only on the status and e-learning supporting strategies at mid-European medical schools, in future studies the focus of analysis should be expanded to meet the conditions in more countries and regions of the world.

## Conclusions

This survey shows that e-learning has gained a firm place in the curricula of those mid-European medical schools addressed. Many institutions have grasped the potential and value of a good infrastructure in this field. However, the distribution and promotion of e-learning is inhomogeneous. Also, teachers’ commitments should be given a better incentive. Aims for the next years should include fostering a network with a constant dialog between the medical schools for addressing common problems, and development of a database with different quality-tested tools that can be accessed on-demand by all schools. This survey should be repeated to document further developments and even extended internationally to compare more countries and to discover potential for future cooperation.

## Additional files

Additional file 1: **The questionnaire, which served as a survey instrument (the text was translated from German into English).** It was made available to the addressed medical schools in Austria, Germany and Switzerland with the online program SurveyMonkey® (SurveyMonkey, Oregon, USA). The survey consisted of 49 items, with 27 closed questions and 22 questions for free text answers. (DOCX 30 kb) Additional file 2: **Original answers** **(*****n*** **= 21)** **of the participating medical schools to the question “Which challenges do you see in the field of e-learning for your medical school in the next years?” (This was translated from German into English.) Each number represents commentaries of one medical school (anonymous).** (DOCX 23 kb)

## References

[1] Ruiz JG, Mintzer MJ, Leipzig RM (2006). The impact of E-learning in medical education. Acad Med..

[2] Xeroulis GJ, Park J, Moulton CA, Reznick RK, Leblanc V, Dubrowski A (2007). Teaching suturing and knot-tying skills to medical students: a randomized controlled study comparing computer-based video instruction and (concurrent and summary) expert feedback. Surgery..

[3] Shantikumar S (2009). From lecture theatre to portable media: students’ perceptions of an enhanced podcast for revision. Med Teach..

[4] Heye T, Kurz P, Eiers M, Kauffmann GW, Schipp A (2008). A radiological case collection with interactive character as a new element in the education of medical students. Rofo..

[5] Ruderich F, Bauch M, Haag M, Heid J, Leven FJ, Singer R (2004). CAMPUS--a flexible, interactive system for web-based, problem-based learning in health care. Stud Health Technol Inform..

[6] Gray K, Tobin J (2010). Introducing an online community into a clinical education setting: a pilot study of student and staff engagement and outcomes using blended learning. BMC Med Educ.

[7] Ridgway PF, Sheikh A, Sweeney KJ, Evoy D, McDermott E, Felle P (2007). Surgical e-learning: validation of multimedia web-based lectures. Med Educ..

[8] Woltering V, Herrler A, Spitzer K, Spreckelsen C (2009). Blended learning positively affects students’ satisfaction and the role of the tutor in the problem-based learning process: results of a mixed-method evaluation. Adv Health Sci Educ Theory Pract..

[9] Back DA, Haberstroh N, Sostmann K, Schmidmaier G, Putzier M, Perka C (2014). High efficacy and students’ satisfaction after voluntary vs mandatory use of an e-learning program in traumatology and orthopedics--a follow-up study. J Surg Educ..

[10] Masie E, Rossett A (2002). Blended learning: The Magic Is in the Mix. The ASTD E-Learning Handbook.

[11] Matthes G, Rixen D, Tempka A, Schmidmaier G, Wolfl C, Ottersbach C (2009). Physicians in traumatology. Critically endangered? Results of an inquiry. Unfallchirurg.

[12] Davids MR, Chikte UM, Halperin ML (2013). An efficient approach to improve the usability of e-learning resources: the role of heuristic evaluation. Adv Physiol Ed..

[13] Steinert Y, Mann K, Centeno A, Dolmans D, Spencer J, Gelula M (2006). A systematic review of faculty development initiatives designed to improve teaching effectiveness in medical education: BEME Guide No. 8. Med Teach.

[14] Welk A, Splieth C, Seyer D, Rosin M, Siemer M, Meyer G (2006). German dental faculty attitudes towards computer-assisted simulation systems correlated with personal and professional profiles. Eur J Dent Educ.

[15] Barefield AC, Meyer JD (2013). Leadership’s role in support of online academic programs: implementing an administrative support matrix. Perspect Health Inf Manag.

[16] German Medical Association. Quality criteria eLearning of the German Medical Association - Qualitätskriterien eLearning der Bundesärztekammer. http://www.bundesaerztekammer.de/fileadmin/user_upload/downloads/KritElearningV8.01.pdf. Accessed 11 Nov 2014

[17] Kim KJ, Han J, Park Ie B, Kee C (2009). Medical education in Korea: the e-learning consortium. Med Teach.

[18] Charité. Quality seal eLearning Charité. http://elearning.charite.de/services/qualitaetssicherung/qualitaetssiegel_elearning/. 2014. Accessed 4 Jan 2014.

[19] St. Amant L, Fechtig L. ‘Structure, Finances and Human Resources’ Working Group Report. University of Toronto, Faculty of Medicine, eLearning task Force. 2015. http://elearning.innovatingedu.ca/wp-content/uploads/2014/09/Structures-Finances-and-HR-WG-Final-Report.pdf. Accessed 29 May 2015.

[20] GMA. https://gesellschaft-medizinische-ausbildung.org (2014). Accessed 16 Sep 2014.

[21] Schnell R, Hill PB, Esser E (2011). Methoden der empirischen Sozialforschung.

[22] Collins D (2003). Pretesting survey instruments: an overview of cognitive methods. Qual Life Res..

[23] Ellaway R, Masters K (2008). AMEE Guide 32: e-Learning in medical education Part 1: Learning, teaching and assessment. Med Teach..

[24] Rasmussen A, Lewis M, White J (2013). The application of wiki technology in medical education. Med Teach..

[25] De Wever B, Van Winckel M, Valcke M (2008). Discussing patient management online: the impact of roles on knowledge construction for students interning at the paediatric ward. Adv Health Sci Educ Theory Pract..

[26] Kowalczyk N, Copley S (2013). Online course delivery modes and design methods in the radiologic sciences. Radiol Technol..

[27] Kolb S, Wengenroth L, Hege I, Praml G, Nowak D, Cantineau J (2009). Case based e-learning in occupational medicine--a European approach. J Occup Environ Med..

[28] Scavo F. Key Advantage of Open Source is Not Cost Savings. 2005. http://www.computereconomics.com/article.cfm?id=1043. Accessed 14 Dec 2014.

[29] Cook DA, Triola MM (2014). What is the role of e-learning? Looking past the hype. Med Educ..

[30] Choules AP (2007). The use of elearning in medical education: a review of the current situation. Postgrad Med J..

[31] Ruiz JG, Candler CS, Qadri SS, Roos BA (2009). E-learning as evidence of educational scholarship: a survey of chairs of promotion and tenure committees at U.S. medical schools. Acad Med.

