# Supplementary material for: Survey of e-learning implementation and faculty support strategies in a cluster of mid-European medical schools
Source: BMC Med Educ. 2015 Sep 3;15:145. doi: 10.1186/s12909-015-0420-4 (PMC4560086; doi:10.1186/s12909-015-0420-4)
Supplement: Additional file 1: — The questionnaire, which served as a survey instrument (the text was translated from German into English). It was made available to the addressed medical schools in Austria, Germany and Switzerland with the online program SurveyMonkey® (SurveyMonkey, Oregon, USA). The survey consisted of 49 items, with 27 closed questions and 22 questions for free text answers. (DOCX 30 kb) [file 12909_2015_420_MOESM1_ESM.docx]

**Survey Questionnaire**

**I. General information about the medical school and provision of e-learning**

1. What is the nearest number of enlisted students at your medical school?

100 – 500, 500-1500, 1500-3000, 3000-6000, 6000-9000, >9000

1. Do e-learning tools exist for the education of students at your medical school?

Yes - predominantly mandatory, Yes - predominantly optional, No

1. Is there a specific set of recommendations for the application of e-learning by your medical school from the academic board or deanery?

Yes/ No

1. Is there a certain budget or other funding sources to support the provision of e-learning offerings at your medical school?

Amount in Euro: No budget, €10.000-50.000, €50.000-100.000, €100.000-250.000, €250.000-400.000, more than €400.000, no comment

1. a. Do you stipulate the use of a quality assurance code for your e-learning activities?

Yes/ No

b. If so, are these quality criteria designed to comply with the standards stipulated by the German Medical Association or similar?

Yes/ No

c. If not – which other quality criteria do you maybe apply? *

Answer:______

d. If you do not apply any quality criteria – why not? *

Answer:______

1. Is there a faculty-wide strategy for increasing the scope and quality of e-learning tools over the coming years in your medical school?

Yes/ No

1. a. How many disciplines at your medical school offer e-learning for the students? *

Answer:______

b. Please name the number of all disciplines at your medical school: *

Answer:______

1. Which challenges do you see in the field of e-learning for your medical school in the next years?

Answer:______

1. Please give in the name of your medical school (optional)

Answer: ______

1. Please give in your faculty position (optional)

Answer: ______

1. Do you take gender criteria into account when developing e-learning activities?

Yes/ No

1. a. Do you wish more support from relevant subject societies (GMA/GMDS etc.) and academic institutions or public agencies (DFG/BMBF/State Ministries) for the development of e-learning activities?

Yes/ No

b. If yes, which kind of support would you like to receive? *

Answer: ______

**II. Information about the infrastructural conditions of e-learning supporting measures**

1. Does your medical school offer
   1. performance-orientated financial rewards (LOM)?

Yes/ No

- 1. specific LOM for teaching?

Yes/ No

- 1. Is e-learning associated with the award of LOM?

Yes/ No

1. a. Do you have permanent staff in your medical school who are employed to deal with e-learning (an e-learning team/department)?

Yes/ No

b. If you do – how many staff members do you have?

Number of staff members:_________

c. If appropriate – how many assisting students do you have?

Number of assisting students:___________

1. a. Do you have a Learning Management System (LMS) at your medical school for providing e-learning offers to your students?

Yes/ More than one / No

b. If you do, what is/are the name/s of the LMS you are using at your medical school?

Answer:_____

Various computer programs ease the development of e-learning contents. The following three questions refer to the use of such kind of software in your institution:

1. Which programs do you offer your teachers for the development and creation of e-learning contents?

Answer:____________

1. Which programs are used alongside those you offer by your teachers on own initiative – as far as you know of?

Answer:__________

1. If you record lectures – which tools do you use?

Answer:______

1. Which e-learning formats/concepts are used at your medical school?

E-learning supplementing a face-to-face course (preparation of scripts or course materials)

Web-based training (optional)

Web-based training (mandatory)

Computer-based training with attendance (optional)

Computer-based training with attendance (mandatory)

Blended learning (optional)

Blended learning (mandatory)

Podcasts/videocasts

Wikis

Webinars

Discussion forums (optional)

Discussion forums (mandatory)

Virtual patients

Digital case collection

Recording of lectures

1. a. Which of the following preclinical disciplines at your medical school offer e-learning? (see Figure 2)

b. Do you have any further preclinical disciplines at your medical school that offer e-learning? *

Answer:______

1. a. Which of the following clinical and other disciplines at your medical school offer e-learning? (see Figure 2)

b. Do you have any further clinical and other disciplines disciplines at your medical school that offer e-learning? *

Answer:______

**III. Information about requirements of and incentives for staff in the field of e-learning**

1. Do you offer training or qualification programs for teachers...
   1. ...that deal directly with the authoring systems of programs in use at your medical school?

Yes/ No

- 1. ...on the topic of e-learning (general information)?

Yes/ No

- 1. Would you make use of training programs that have been developed at other universities for the training of your teachers?

Yes/ No

1. Does your medical school use electronic means to carry out summative (mandatory) exams?

Yes/ No

1. Does your medical school also offer e-learning formative exams to students?

Yes/ No

1. Do you reward your teachers in some form for...
   1. … the development of e-learning tools/courses?

Yes/ No

- 1. …the implementation of e-learning tools/courses?

Yes/No

- 1. If you do – which kind of awards do you use (performance-orientated financial rewards, prizes of the faculty, quality seals, etc.)?

Answer:_____________

1. a. Do development and implementation of e-learning tools/courses count towards teaching activities or load?

Yes/ No

b. If they do, according to which regulations?

Answer:_________

1. If not, is this planned at your medical school for the future?

Yes/ No

1. Would you consider offering e-learning opportunities developed by other medical schools for inclusion in teaching by your medical school, if they fitted into the curriculum?

Yes/ No

1. Are teachers at your medical school encouraged (through instructions, study regulations, etc.) to prepare e-learning tools?

Yes/ No

1. Do you regularly evaluate the opinions, attitudes and experiences of your teachers on the subject of e-learning in your medical school?

Yes/ No

1. Do you regularly evaluate the opinions, attitudes and experiences of your students on the subject of e-learning in your medical school?

Yes/ No

1. Does your medical school or do your students recognise outstanding e-learning opportunities with awards?

Yes/ No

-----------------------------

* Answers to these questions were not included in the analysis and the corresponding manuscript as there were less than five responses or a high ambiguity among the answers given that inhibited an analysis.
