# Supplementary material for: Survey of e-learning implementation and faculty support strategies in a cluster of mid-European medical schools
Source: BMC Med Educ. 2015 Sep 3;15:145. doi: 10.1186/s12909-015-0420-4 (PMC4560086; doi:10.1186/s12909-015-0420-4)
Supplement: Additional file 2: — Original answers (n = 21) of the participating medical schools to the question “Which challenges do you see in the field of e-learning for your medical school in the next years?” (This was translated from German into English.) Each number represents commentaries of one medical school (anonymous). (DOCX 23 kb) [file 12909_2015_420_MOESM2_ESM.docx]

# Additional file 2: E-learning challenges for medical schools

Twenty-one medical schools listed the following e-learning challenges:

| **No.** | **Challenges listed** |
| --- | --- |
| 1 | - Adaptation and implementation of the existing e-learning concepts into new study tracks. - Representation of the efforts to produce e-learning features. |
| 2 | - - Overview of all e-learning offers in the medical school – to communicate the additional value to both teachers and students.   - Developing a strategy about e-learning for the entire medical school.   - Integration of existing e-learning proposals into the curriculum.   - Conception of the offers according to the learning objectives.   - Designing presentations so that they are interactive and attractive. |
| 3 | - - Better structural embedding.   - Clear responsibilities.   - Missing personnel resources.   - Dealing with unclear regulations about accounting for the teachers' load/incentives. |
| 4 | - - Extension of the existing offers.   - Information and trainings of the teachers.   - Finding funding sources. |
| 5 | - Adequate personnel (at present only one 20% full-time equivalent for e-learning). |
| 6 | - - Utilizing the existing means more efficiently.   - Improving infrastructure. |
| 7 | - - Including teachers and students.   - Financing.   - Proof of benefit and efficacy of e-learning. |
| 8 | - E-learning should consist of more than merely using the LMS – which is the case right now. A meaningful integration of digital teaching and learning formats into an existing curriculum as “blended learning” is a huge challenge. It demands expertise, resources, and the willingness of the teachers to think actively about those formats for any further development in the right direction. |
| 9 | - - Introduction of interactive e-learning tools. Until now, most learning materials are available merely for download.   - There are only few projects where e-learning is integrated into the curricular concept.   - Furthermore, e-learning is only rarely accepted as being part of the teaching load, and this is something which reduces the motivation of the teaching staff to use it. Specially made systems which are incentive and/or rewarding here have to be designed to address this issue. |
| 10 | - Incorporating the use of smartphones and/or social media. |
| 11 | - - To introduce e-learning as part of the curricular concept in the form of blended learning, not just as an additional “nice-to-have thing”.   - To apply e-learning in a way that students can get an individual feedback.   - To develop e-learning concepts which are close to reality.   - To improve and update e-learning platforms continuously.   - To motivate their teachers to use e-learning in a meaningful way. |
| 12 | - - Adoption to the ongoing reform of the study track which will we based on modules with orientation to symptoms instead of certain disciplines.   - Uncertainties concerning copyright problems. |
| 13 | - - To increase the acceptance of using e-learning among the teachers.   - To explain its use to the teaching staff.   - To realize the development of e-learning offerings (i.e. to win the teachers for cooperating in developing new offers). |
| 14 | - Successful integration of e-learning into face-to-face teaching. |
| 15 | - Developing a strategy for the usage of e-learning in the whole medical school. |
| 16 | - - Use of the learning management system within the entire medical school – as far as possible.   - Introduction of modern scenarios (e.g. blended learning). |
| 17 | - - Media didactics.   - Copyright issues.   - Continuation. |
| 18 | - Finding qualified personnel. |
| 19 | - - Teaching the content provider.   - Enabling accessibility to the contents via various platforms (tablets, smartphones etc.). |
| 20 | - Creation of contents and school-spanning exchange (e.g. lecture recordings). |
| 21 | - Missing IT competence in the faculty and the necessary high support. |
